# Supplementary material for: A Functional Pipeline of Genome-Wide Association Data Leads to Midostaurin as a Repurposed Drug for Alzheimer’s Disease
Source: Int J Mol Sci. 2023 Jul 28;24(15):12079. doi: 10.3390/ijms241512079 (PMC10418421; doi:10.3390/ijms241512079)
Supplement: Supplementary file 1 [file ijms-24-12079-s001.zip › Supplementary Table S1.pdf]

Supplementary Table S1. GO terms over- and under-represented in the STRING target gene network. ENR: strength of enrichment. FDR: False Discovery Rate (calculation includes the Benjamini-Hochberg correction).

	GO Term	ENR	FDR
**Under-represented**	Membrane	0.09	0.048
	Organelle membrane	0.21	0.024
	Cytoplasmic vesicle	0.22	0.043
	Bounding membrane of organelle	0.28	0.024
	Whole membrane	0.30	0.024
	Endosome	0.36	0.031
	Golgi membrane	0.36	0.043
	Cytoplasmic vesicle membrane	0.36	0.048
	Vesicle membrane	0.37	0.038
	Presynapse	0.43	0.043
	Transport vesicle	0.48	0.036
	Early endosome	0.48	0.043
	Organelle subcompartment	0.50	0.031
	Golgi apparatus subcompartment	0.50	0.038
	Integral component of organelle membrane	0.53	0.031
	trans-Golgi network	0.55	0.048
	Synaptic vesicle	0.59	0.048
	Regulation of endocytosis	0.78	0.003
	Ficolin-1-rich granule membrane	0.94	0.031
**Over-represented**	Protein-lipid complex	1.11	0.024
	Negative regulation of endocytosis	1.15	0.090
	Alzheimer's disease	1.18	0.027
	High-density lipoprotein particle	1.18	0.024
	Triglyceride-rich plasma lipoprotein particle	1.22	0.031
	Spherical high-density lipoprotein particle	1.47	0.035
