# Supplementary material for: A Functional Pipeline of Genome-Wide Association Data Leads to Midostaurin as a Repurposed Drug for Alzheimer’s Disease
Source: Int J Mol Sci. 2023 Jul 28;24(15):12079. doi: 10.3390/ijms241512079 (PMC10418421; doi:10.3390/ijms241512079)
Supplement: Supplementary file 1 [file ijms-24-12079-s001.zip › Supplementary Table S2.pdf]

Supplementary Table S2. DAGGER summary table

<table>
<tr><th>GWAS</th><th colspan="2">GTEx</th><th>DGIdb</th></tr>
<tr><td rowspan="2">Total polymorphisms:
16 358 697</td><td>GTEx genes:
39 680</td><td>Max Q-value:
0.00186873</td><td rowspan="2">Druggable targets: 69</td></tr>
<tr><td>GTEx eQTL:
1 208 024</td><td>Significant AD eQTL:
498</td></tr>
<tr><td rowspan="2">Selected p-value:
0.001</td><td>Q-value cutoff: 5%</td><td>Random AD eQTL:
135</td><td rowspan="3">Treatable targets: 22</td></tr>
<tr><td>Filtered genes:
21 057</td><td>Final AD eQTL:
437 (61 matches)</td></tr>
<tr><td>Filtered
polymorphisms:
41 919</td><td>Filtered eQTL: 60
401</td><td>Potential targets: 298</td></tr>
</table>
