# Supplementary material for: A Functional Pipeline of Genome-Wide Association Data Leads to Midostaurin as a Repurposed Drug for Alzheimer’s Disease
Source: Int J Mol Sci. 2023 Jul 28;24(15):12079. doi: 10.3390/ijms241512079 (PMC10418421; doi:10.3390/ijms241512079)
Supplement: Supplementary file 1 [file ijms-24-12079-s001.zip › SupplementaryLegends.pdf]

Supplementary Figure S1. Bar plots showing the distribution of selected SNPs (A) and matched eQTL (B) in the significant (blue) and randomly selected (black) datasets. Volcano plots showing the distribution of p-values vs. odds ratios in the statistically significant (C) and random (D) SNP datasets. The horizontal line shown in each Volcano Plot corresponds to a p-value = 10-3.

Supplementary Figure S2. Independent assays showing the effect Midostaurin on *C. elegans* GMC101. Two independent repetitions of the motility experiment with Midostaruin at 50 mM are plot. Mantel-Cox assays for both cases were p = 0.089 (A), and p = 0.012 (B). N ≥ 60

Supplementary Table S1. GO terms over- and under-represented in the STRING target gene network. ENR: strength of enrichment. FDR: False Discovery Rate (calculation includes the Benjamini-Hochberg correction).

Supplementary Table S2. DAGGER summary table. GWAS section describes total SNPs, p-value filter and number of SNPs after filtering. GTEx section includes total genes and eQTL in the database, top % of Q-value eQTL to select, and number of genes and eQTL after selection. Also includes max value of Q after selection. Significant and random AD eQTL refer to the GWAS SNP with matches in the top GTEx eQTL respectively. Final AD eQTL include the final number of eQTL, after subtracting matches from the random dataset. Potential targets are genes identified after final eQTL matching. Druggable targets: potential targets affected by known drugs according to DGIdb. Treatable targets: potential targets whose associated drug has a beneficial effect according to DAGGER prediction.

Supplementary Table S3. DAGGER results table including GWAS imput results, GTEx described effects on gene expression, and drug information from DGIDb. Gene_id: ENSEMBL gene identifier. Ref: reference allele. Alt: alternate allele. Rs_id: SNP ID. MAF: Minor Allele Frequency. Slope: size effect of the SNP on gene expression. A positive slope means expression is increased, a negative slope means it is decreased. Beta: $\ln(Odds\ Ratio)$. SE: standard error of the beta. P: p-value of beta.

Supplementary Table S4. Results of gprofileR2 structural analysis on significant GWAS SNPs. Of the 4183 unique genes shown in this table, 204 are also identified by DAGGER.

Supplementary Table S5. Results of gprofileR2 structural analysis on random SNPs selected from GWAS results. Of the 11011 unique genes shown in this table, 53 are also identified by DAGGER.
