# Supplementary figures and images for: A Functional Pipeline of Genome-Wide Association Data Leads to Midostaurin as a Repurposed Drug for Alzheimer’s Disease
Source: Int J Mol Sci. 2023 Jul 28;24(15):12079. doi: 10.3390/ijms241512079 (PMC10418421; doi:10.3390/ijms241512079)

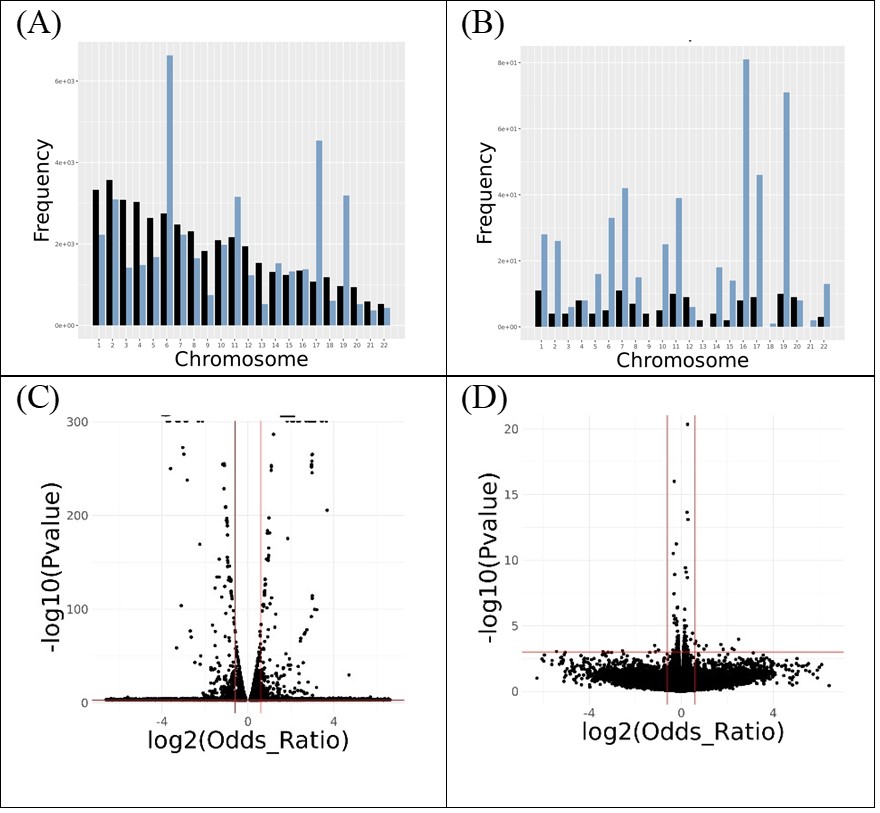

Supplement: Supplementary file 1 [file ijms-24-12079-s001.zip › Figure S1.jpg]

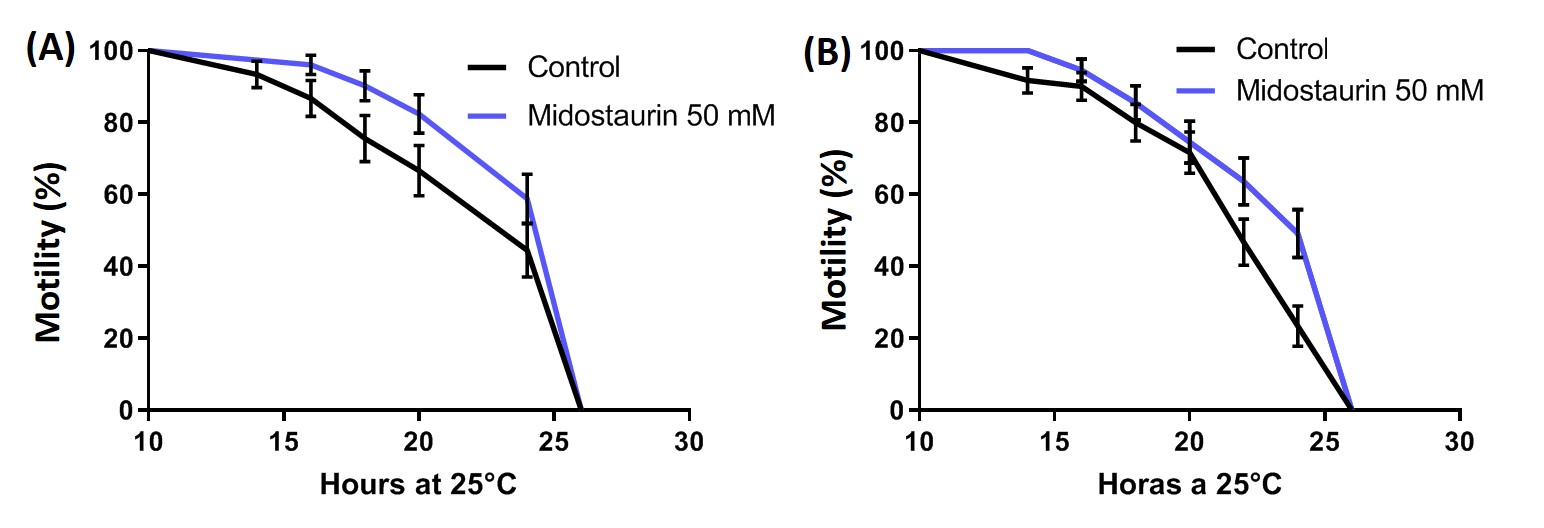

Supplement: Supplementary file 1 [file ijms-24-12079-s001.zip › Figure S2_revised.jpg]
